# Supplementary material for: Modeling glioblastoma heterogeneity as a dynamic network of cell states
Source: Mol Syst Biol. 2021 Sep 16;17(9):e10105. doi: 10.15252/msb.202010105 (PMC8444284; doi:10.15252/msb.202010105)
Supplement: Supplementary file 6 — Source Data for Figure 5 [file MSB-17-e10105-s004.zip › Figure5A_sourcedata/GSEA_3071/hallmarks_stateVI.GseaPreranked.1623742557031/HALLMARK_MTORC1_SIGNALING.html]

Details for gene set HALLMARK\_MTORC1\_SIGNALING[GSEA]

|  || Dataset | state63071 |
| Phenotype | NoPhenotypeAvailable |
| Upregulated in class | na\_pos |
| GeneSet | HALLMARK\_MTORC1\_SIGNALING |
| Enrichment Score (ES) | 0.24387687 |
| Normalized Enrichment Score (NES) | 1.6799456 |
| Nominal p-value | 0.010416667 |
| FDR q-value | 0.041844834 |
| FWER p-Value | 0.143 |
Table: GSEA Results Summary

  

Fig 1: Enrichment plot: HALLMARK\_MTORC1\_SIGNALING      
 Profile of the Running ES Score & Positions of GeneSet Members on the Rank Ordered List

  

| PROBE | GENE SYMBOL | GENE\_TITLE | RANK IN GENE LIST | RANK METRIC SCORE | RUNNING ES | CORE ENRICHMENT || 1 | DDIT3 |  |  | 14 | 1.086 | 0.0265 | Yes |
| 2 | PPP1R15A |  |  | 98 | 0.738 | 0.0239 | Yes |
| 3 | PNO1 |  |  | 144 | 0.672 | 0.0301 | Yes |
| 4 | IGFBP5 |  |  | 145 | 0.671 | 0.0489 | Yes |
| 5 | SQSTM1 |  |  | 147 | 0.667 | 0.0673 | Yes |
| 6 | LDHA |  |  | 197 | 0.604 | 0.0705 | Yes |
| 7 | ENO1 |  |  | 223 | 0.587 | 0.0799 | Yes |
| 8 | PSMG1 |  |  | 246 | 0.568 | 0.0897 | Yes |
| 9 | GLRX |  |  | 330 | 0.523 | 0.0810 | Yes |
| 10 | TRIB3 |  |  | 387 | 0.495 | 0.0792 | Yes |
| 11 | XBP1 |  |  | 389 | 0.494 | 0.0927 | Yes |
| 12 | HSPA5 |  |  | 414 | 0.484 | 0.0996 | Yes |
| 13 | EEF1E1 |  |  | 459 | 0.468 | 0.1003 | Yes |
| 14 | PSMC4 |  |  | 472 | 0.463 | 0.1100 | Yes |
| 15 | ACTR3 |  |  | 486 | 0.459 | 0.1192 | Yes |
| 16 | ATP5MC1 |  |  | 494 | 0.457 | 0.1300 | Yes |
| 17 | MAP2K3 |  |  | 550 | 0.440 | 0.1269 | Yes |
| 18 | POLR3G |  |  | 558 | 0.437 | 0.1372 | Yes |
| 19 | AURKA |  |  | 572 | 0.432 | 0.1457 | Yes |
| 20 | PPA1 |  |  | 594 | 0.427 | 0.1517 | Yes |
| 21 | PNP |  |  | 625 | 0.418 | 0.1550 | Yes |
| 22 | PSMD13 |  |  | 683 | 0.404 | 0.1504 | Yes |
| 23 | SYTL2 |  |  | 707 | 0.395 | 0.1550 | Yes |
| 24 | YKT6 |  |  | 711 | 0.395 | 0.1652 | Yes |
| 25 | ETF1 |  |  | 715 | 0.393 | 0.1754 | Yes |
| 26 | BCAT1 |  |  | 791 | 0.378 | 0.1649 | Yes |
| 27 | ME1 |  |  | 818 | 0.374 | 0.1681 | Yes |
| 28 | ATP6V1D |  |  | 822 | 0.372 | 0.1777 | Yes |
| 29 | NFKBIB |  |  | 864 | 0.366 | 0.1765 | Yes |
| 30 | ARPC5L |  |  | 868 | 0.366 | 0.1859 | Yes |
| 31 | RRP9 |  |  | 872 | 0.365 | 0.1953 | Yes |
| 32 | CCT6A |  |  | 902 | 0.361 | 0.1972 | Yes |
| 33 | GOT1 |  |  | 931 | 0.356 | 0.1993 | Yes |
| 34 | DDX39A |  |  | 936 | 0.355 | 0.2082 | Yes |
| 35 | SDF2L1 |  |  | 947 | 0.353 | 0.2153 | Yes |
| 36 | TOMM40 |  |  | 953 | 0.352 | 0.2237 | Yes |
| 37 | NFIL3 |  |  | 956 | 0.352 | 0.2330 | Yes |
| 38 | TPI1 |  |  | 972 | 0.350 | 0.2386 | Yes |
| 39 | SLC7A5 |  |  | 989 | 0.348 | 0.2439 | Yes |
| 40 | IFRD1 |  |  | 1085 | 0.333 | 0.2265 | No |
| 41 | HSPA9 |  |  | 1087 | 0.333 | 0.2356 | No |
| 42 | PGK1 |  |  | 1099 | 0.331 | 0.2418 | No |
| 43 | SLC1A5 |  |  | 1169 | 0.320 | 0.2314 | No |
| 44 | G6PD |  |  | 1170 | 0.320 | 0.2403 | No |
| 45 | ABCF2 |  |  | 1214 | 0.314 | 0.2371 | No |
| 46 | SORD |  |  | 1239 | 0.311 | 0.2390 | No |
| 47 | STIP1 |  |  | 1311 | 0.302 | 0.2276 | No |
| 48 | HMBS |  |  | 1315 | 0.301 | 0.2352 | No |
| 49 | GTF2H1 |  |  | 1348 | 0.298 | 0.2345 | No |
| 50 | UBE2D3 |  |  | 1361 | 0.296 | 0.2395 | No |
| 51 | SERP1 |  |  | 1428 | 0.288 | 0.2290 | No |
| 52 | HSPD1 |  |  | 1556 | 0.274 | 0.2010 | No |
| 53 | SLC2A1 |  |  | 1606 | 0.268 | 0.1948 | No |
| 54 | PDK1 |  |  | 1609 | 0.268 | 0.2017 | No |
| 55 | PSMC2 |  |  | 1646 | 0.264 | 0.1990 | No |
| 56 | PLK1 |  |  | 1665 | 0.263 | 0.2014 | No |
| 57 | PPIA |  |  | 1715 | 0.259 | 0.1949 | No |
| 58 | CTSC |  |  | 1767 | 0.254 | 0.1877 | No |
| 59 | NUFIP1 |  |  | 1770 | 0.254 | 0.1942 | No |
| 60 | RPA1 |  |  | 1849 | -0.256 | 0.1795 | No |
| 61 | UNG |  |  | 2284 | -0.330 | 0.0669 | No |
| 62 | CCNF |  |  | 2350 | -0.343 | 0.0583 | No |
| 63 | IDH1 |  |  | 2526 | -0.384 | 0.0199 | No |
| 64 | ELOVL6 |  |  | 2575 | -0.392 | 0.0174 | No |
| 65 | MLLT11 |  |  | 2850 | -0.476 | -0.0462 | No |
| 66 | PIK3R3 |  |  | 2903 | -0.495 | -0.0469 | No |
| 67 | FADS2 |  |  | 3173 | -0.640 | -0.1045 | No |
| 68 | MCM4 |  |  | 3254 | -0.715 | -0.1069 | No |
| 69 | CXCR4 |  |  | 3302 | -0.759 | -0.0988 | No |
| 70 | DHFR |  |  | 3303 | -0.765 | -0.0774 | No |
| 71 | RRM2 |  |  | 3317 | -0.783 | -0.0591 | No |
| 72 | TM7SF2 |  |  | 3334 | -0.802 | -0.0411 | No |
| 73 | NUPR1 |  |  | 3366 | -0.835 | -0.0264 | No |
| 74 | BTG2 |  |  | 3377 | -0.857 | -0.0052 | No |
| 75 | STC1 |  |  | 3393 | -0.880 | 0.0152 | No |
| 76 | CTH |  |  | 3401 | -0.894 | 0.0384 | No |
| 77 | CD9 |  |  | 3445 | -0.995 | 0.0542 | No |
Table: GSEA details [plain text format]

  

Fig 2: HALLMARK\_MTORC1\_SIGNALING: Random ES distribution      
 Gene set null distribution of ES for **HALLMARK\_MTORC1\_SIGNALING**
